# Leptospirosis: Skin Wounds and Control Strategies, Thailand, 1999

**DOI:** 10.3201/eid0812.020180

**Published:** 2002-12

**Authors:** Phran Phraisuwan, Ellen A. Spotts Whitney, Piyanit Tharmaphornpilas, Suriya Guharat, Samart Thongkamsamut, Suphaporn Aresagig, Jayteeya Liangphongphanthu, Kanlayanee Junthima, Apirat Sokampang, David A. Ashford

**Affiliations:** * Ministry of Public Health, Nonthaburi, Thailand; † Centers for Disease Control and Prevention, Atlanta, Georgia, USA; ‡ Buriram Provincial Health Office, Buriram, Thailand; § Khumuang hospital, Buriram, Thailand; and ¶Northeastern Regional Epidemiology Center, Nakornratchasima, Thailand

**Keywords:** Leptospirosis, *Leptospira*, skin wounds, control strategies, Thailand

## Abstract

After an outbreak of leptospirosis in workers who participated in cleaning a pond during September 1999 in Thailand, a serologic survey was conducted. Among a cohort of 104 persons from one village who participated in pond cleaning activity, 43 (41.3%) were seropositive for immunoglobulin M antibodies against *Leptospira*, indicating recent infection. Only 17 (39.5%) of 43 seropositive persons reported a recent febrile illness; the remaining seropositive persons were considered asymptomatic, suggesting that asymptomatic leptospirosis infection may be common where leptospirosis is endemic. Multivariable logistic regression indicated that wearing long pants or skirts was independently protective against leptospirosis infection (OR_adjusted_ = 0.217), while the presence of more than two wounds on the body was independently associated with infection (OR_adjusted_ = 3.97). Educational efforts should be enhanced in areas where leptospirosis is endemic to encourage the use of protective clothing. In addition wound management and avoidance of potentially contaminated water when skin wounds are present should be included in health education programs.

Leptospirosis, a worldwide zoonotic disease, is caused by spirochetes of the genus *Leptospira*. In Thailand, a nationwide leptospirosis epidemic is ongoing and control strategies are being explored (1–5). The number of cases reported from 1982 to 1995 ranged from 55 to 272 cases per year, with an average incidence of 0.3/100,000/year (5). The number of leptospirosis cases reported in 1996 was 398 (incidence 0.65/100,000); the number of cases in 1997 was 2,334 (3.83/100,000). In 1998, the number of cases was 2,230 (3.52100,000), in 1999, the number of cases was 6,080 (9.89/100,000), and in 2000, the number of cases was 14,286 (23.2//100,000) (6). In Thailand, leptospirosis corresponds with the rainy season, with an increase in cases beginning in August and decreasing in November; the peak number of cases occurs in October (6).

Surveillance data suggest that most infections occur in agricultural workers, primarily rice producers (1–5). Infection in humans occurs through contact of skin or mucous membranes with water or moist soil contaminated with urine of infected animals (7–10). Breaks in skin in facilitate infection, but no previous study has quantified the correlation between skin wounds and leptospirosis (11,12). Heavy rainfall and flooding; going without shoes; washing in streams; and occupations such as farming, working in sewers, mining, working with animals, and participating in military activities have all been implicated in human infection (7,10,13–19). Despite identification of these risk factors, control strategies for leptospirosis are lacking.

 In September and October 1999, an outbreak of leptospirosis occurred in the Khumuang subdistrict of Buriram, a province in northeastern region of Thailand. No cases of leptospirosis had been reported in this subdistrict for the previous 2 years. Local health officers from the Khumuang Hospital notified the Khumuang District Health Office of an abnormal increase in numbers of patients meeting the World Health Organization (WHO) criteria for leptospirosis infection; 80 cases of leptospirosis were identified from September 19 to 29, 1999.

In association with the outbreak, we conducted a study of persons who participated in pond cleaning activities and used a nested case-control study to compare participants with and without leptospiosis infection. Pond cleaning activities included entering the water, pulling up foliage, and removing debris. The objectives of the study were to 1) estimate the attack rate among pond cleaners in this setting, 2) determine risk factors for leptospirosis infection, and 3) identify possible control and prevention strategies. The results of this study, including the attack rate of leptospirosis, the association between modifiable risk factors for leptospirosis and infection, and the prevalence of asymptomatic infection are reported.

## Materials and Methods

### Study Design and Method

A cross-sectional survey of pond cleaning participants was conducted from October 6 to 8, 1999, in the subdistrict of Khumuang, Buriram Province, Thailand. The survey was conducted with a convenience sample of 315 persons from the total number who participated in the pond cleaning (n=500). All 315 persons were part of an agricultural community located in the Khumuang subdistrict, approximately 400 km from Bangkok and 100 km from Thai-Cambodian border.

Twelve teams, each consisting of three or four health-care providers (doctor or nurse, health officer, and health volunteer), visited this village from October 6 to 8, 1999. All 315 persons from the village involved in the cleaning of Nong Tad were asked to participate in this study. Two hundred twenty-eight (72%) workers consented to be interviewed and were given a standardized questionnaire. Questions included demographics and risk factor information, such as the working site in the pond, lunch eating site, clothing worn while working, other probable exposure to leptospirosis, and presence of wounds during their participation in the cleaning activity. If a participant answered yes to having skin wounds during the pond cleaning activity, his or her body was examined for lesions or scars. and the interviewer subsequently noted the affected body parts. Participants were asked if they had been ill since participating in the pond cleaning activity. Illness was defined as having symptoms meeting the WHO criteria for leptospirosis (20). Clinical information was collected through review of medical records for those participants who reported being ill and who were seen at the Khumuang Hospital. Serum samples were obtained 1 week after the interview from 104 (45.6%) of the 228 participants; the other 124 persons refused to undergo phlebotomy. Therefore, the interviewer did not know the seropositivity status of the participants at the time of the interview. The timing of the serum collections was within 1 month after exposure to the pond. Infected and noninfected pond cleaning participants were evaluated for risk factors for infection in a nested case-control study. All specimens were tested in the Thailand Ministry of Health Laboratories by using the Lepto-Dipstick Test (Organon, Dublin, Ireland), a commercial test kit with sensitivity and specificity exceeding 80% (21). This study was determined to be a public health response that did not require IRB review.

### Case Definition

A case was defined as the presence of immunoglobulin (Ig) M anti-leptospiral antibodies by the Lepto-Dipstick Test in a person from the Khumuang subdistrict who participated in the survey. Persons with a positive IgM antibody response were considered to have incident cases because the serum was collected and tested 1 month after exposure and IgM antibodies last an average of 3–6 months (22–24). Asymptomatic infection was defined as a positive IgM response in a person who did not report having fever, myalgias, headache, or other evidence of leptospirosis.

### Statistical Analysis

Descriptive statistics and subsequent multivariable analysis were derived through the use of SAS software release 8.1 (SAS Institute, Inc., Cary, NC). All variables were dichotomized except for age, which was treated as a continuous variable.

Demographic, environmental, and behavioral exposure variables were compared for infected and noninfected persons by univariate analyses. All two-way interactions between variables were tested. A multivariable model was created by inclusion of all exposures significant by univariate analysis as well as age and sex to control for confounding, and a backward elimination procedure was performed to identify exposure variables most strongly associated with seropositivity for leptospirosis infection. Confounding in the absence of interaction was assessed by comparing odds ratios (OR) of the exposure variables in the gold standard model controlling for the covariates, age and sex, with the odds ratios of the exposure variables in the reduced models without age and sex, respectively. If a difference of >10% between the OR was detected, confounding was present and the covariate was retained in the model (25). The variance-covariance matrix allowed for the calculation of 95% confidence intervals (CI) for the OR involving the estimated coefficient of any significant interaction term. A SAS macro was used to calculate the conditional indices and variance decomposition proportions, allowing for the assessment of multicolinearity for two or more variables.

## Results

Blood samples were collected and tested from 104 (45.6%) of 228 pond cleaning participants for serologic testing. The subset of 104 persons who agreed to participate in the serosurvey was similar to nonparticipants in the distribution of age (p=0.387) and sex (p=0.124). In addition, all 228 participants reported farming as their occupation. The serologic survey population consisted of 55 men and 49 women with a median age of 38.5 years (range 15–65).

Of the 104 serum samples tested by the Lepto-Dipstick Test, 43 were seropositive for IgM antibodies against *Leptospira*, indicating recent leptospirosis infection (attack rate=41.3%).

### Infection Attack Rate and Asymptomatic Infection

Of the 43 persons with IgM anti-*Leptospira* antibodies, only 17 (39.5%) reported having illness that met the WHO criteria for leptospirosis; the remaining 26 (60.5%) had asymptomatic infection. Clinical information was available for 13 (76.5%) of the 17 infected persons whose illness met the WHO criteria for leptospirosis. Four people did not seek treatment at Khumuang Hosptial for their illness; their clinical information was not available. All 13 persons who sought treatment at Khumuang Hospital had a fever, 10 above 39°C. Other predominant clinical presentations included chills 84.6% (11/13), headache 76.9% (10/13), myalgia 84.6% (11/13), and calf pain 76.9% (10/13).

### Descriptive Statistics and Univariate Logistic Regression

Univariate associations between exposures and leptospiral infection among the 104 persons sampled are presented in Table 1. The median age of infected persons (35, range 15–65) was not significantly different from the median age of noninfected persons (40, range 15–63). Although the infection rate in women (32.7%) was lower than that of men (62.8%), the difference was not statistically significant (p=0.091).

**Table 1:** Results of univariate analysis of potential risk factors for leptospirosis infection among persons participating in cleaning a pond: odds ratios (OR), 95% confidence intervals (CI), and chi-square p value

Risk Factor	Infected (n = 43) (%)		Noninfected (n = 61) (%)		OR (95%CI)	p value
Demographic						
Gender						
Male	27 (62.8)	28 (45.9)		1.99 (0.896 to 4.42)		0.091
Female	16 (37.2)	33 (54.1)				
Age in yrs (continuous)				0.970 (0.939 to 1.00)		
Individual						
Reported clinical illness	17 (39.5)	15 (24.6)		2.01 (0.862 to 4.67)		0.106
Location in the pond where work was preformed						
Site 1	5 (11.6)	10 (16.4)		0.67 (0.212 to 2.13)		0.498
Site 2	13 (30.2)	14 (23.0)		1.45 (0.602 to 3.52)		0.405
Site 3	15 (34.9)	30 (49.2)		0.55 (0.248 to 1.24)		0.149
Site 4	11 (25.6)	17 (27.9)		0.89 (0.367 to 2.16)		0.796
Site 5	14 (32.6)	14 (23.0)		1.62 (0.677 to 3.88)		0.279
Site 6	7 (16.3)	7 (11.5)		1.50 (0.485 to 4.64)		0.482
Site 7	10 (23.3)	11 (18.0)		1.38 (0.526 to 3.61)		0.514
Place where person ate lunch, pond rim vs. elsewhere	2 (4.7)	5 (8.2)		0.55 (0.101 to 2.96)		0.483
Clothing worn while working in the pond						
Shirt	443 (100)	60 (98.4)		*		0.986
Short sleeve shirt	10 (23.3)	7 (11.5)		2.34 (0.811 to 6.74)		0.116
Long sleeve shirt	33 (76.7)	53 (86.9)		0.500 (0.178 to 1.39)		0.183
Trousers	26 (60.5)	44 (72.1)		0.590 (0.258 to 1.35)		0.214
Long skirt	6 (14.0)	11 (18.0)		0.740 (0.250 to 2.17)		0.581
Trousers or long skirt vs. shorts	32 (74.4)	55 (90.2)		0.32 (0.107 to 0.940)		0.038
Any type of glove	1 (2.3)	2 (3.3)		0.700 (0.062 to 8.00)		0.776
Slippers	5 (11.6)	11 (18.0)		0.600 (0.192 to 1.87)		0.376
Tennis shoes or cut shoes	1 (2.3)	1 (1.6)		1.43 (0.087 to 23.5)		0.803
Boots	2 (4.7)	7 (11.5)		0.38 (0.074 to 1.91)		0.238
Boots filled with water	3 (7.0)	12 (19.7)		0.310 (0.081 to 1.16)		0.082
Any use of footwear	11 (25.6)	31 (50.8)		0.33 (0.142 to 0.778)		0.011
Wounds present while working in the pond						
Any hand wound	31 (72.1)	34 (55.7)		2.05 (0.889 to 4.73)		0.092
Hand wounds, 0–5 vs. 6 or more	12 (27.9)	11 (18.0)		1.76 (0.692 to 4.47)		0.235
Any trunk wound	2 (4.7)	2 (3.3)		1.44 (0.195 to 10.6)		0.721
Trunk wounds, 0–5 vs. 6 or more	1 (2.3)	1 (1.6)		1.43 (0.087 to 23.5)		0.803
Any leg wounds	10 (23.3)	6 (9.8)		2.78 (0.924 to 8.35)		0.069
Leg wounds, 0–5 vs. 6 or more	5 (11.6)	1 (1.6)		7.89 (0.888 to 70.2)		0.064
Any foot wound	3 (7.0)	9 (14.8)		0.43 (0.110 to 1.71)		0.232
Any wound	33 (76.7)	40 (65.6)		1.73 (0.716 to 4.19)		0.233
Total number of wounds dichotomized at the median, 2.0	21 (48.8)	13 (21.3)		3.52 (1.50, 8.30)		0.004

In univariate analysis, the pond sites where people worked and places where lunch was eaten were not associated with infection. Infection was not significantly associated with the presence of hand wounds (p=0.092), leg wounds (p=0.069), or six or more leg wounds (p=0.064). Yet having more than two wounds anywhere on the body was significantly associated with infection (p=0.004). Additionally, wearing trousers or long skirts was significantly protective against infection (p=0.038). Trousers or long skirts were worn by 48 (98%) of 49 women and 39 (70.9%) of 55 men (p=0.0002), and footwear was worn by 30 (61.2%) of 49 women and 12 (21.8%) of 55 men (p<0.0001).

### Multivariate Logistic Regression

All variables associated with or protective against leptospirosis infection with a p value <0.1 by univariate analysis were included in the multivariable logistic regression model. No interaction was detected between any of the exposures. Age was retained in the model throughout the backwards elimination procedure to control for confounding. Sex did not confound any variables that remained in the model after backwards elimination and was removed from the final model. Although any type of footwear was protective in univariate, it was not independent in multivariable analysis. Multivariable analysis by using a backwards elimination procedure (p<0.05) while controlling for age indicated that having a total of more than two wounds anywhere on the body while working in the pond remained independently associated with infection (OR_adjusted_=3.97, 95% CI 1.56 to 10.2), while wearing trousers or long skirts was protective (OR_adjusted_=0.23, 95% CI 0.067 to 0.701) (Table 2). Multicolinearity was not detected among any of the variables in the multivariate model.

**Table 2:** Risk factors for leptospirosis infection by multivariable logistic regression controlling for age: odds ratios (OR), 95% confidence intervals (CI), and chi-square p values.

Variable	Adjusted OR	95% CI	p value
Age in yrs	0.980	(0.947 to 1.01)	0.247
Total number of wounds dichotomized at the median, 2.0	3.97	(1.56 to 10.1)	0.004
Long trousers or skirts vs. shorts	0.217	(0.067 to 0.701)	0.011

## Discussion

Symptomatic *Leptospira* infection is often characterized as febrile illness accompanied by other symptoms including headache, conjunctival suffusion, muscle pains, and meningismus (26,27). Yet some persons may have clinically inapparent infection or symptoms too mild to be definitively diagnosed, especially in disease-endemic areas (7,28). The advantage of using the Lepto-Dipstick Test lies in its ability to detect serum IgM antibodies against *Leptospira*, showing recent infection. Studies have shown that antibodies against *Leptospira* develop 4–6 days after exposure and can be detected 3–6 months after illness; however, the length of persistence of the IgM antibodies is unknown (10,22–24). Based on this test, our findings suggest that the proportion of asymptomatic infection for leptospirosis was 60.5% in this population. The asymptomatic infection rate reported here is consistent with other studies, which have shown asymptomatic infection rates up to 70.6% (19,27–30). Tangkanakul et al. reported a background asymptomatic infection rate of 8.4%–11% in a disease-endemic area of Thailand from August to December 1998 (31). This background rate is much lower than the asymptomatic infection rate that we found in our population, which may indicate that the pond may be the source of infection rather than some other reservoir. However, misclassification of these seropositive persons may be a potential bias. Persons with asymptomatic infection are unlikely to be important in the transmission of leptospirosis, since person-to-person transmission is known to be rare in symptomatic patients (9,32). The full importance of subclinical or asymptomatic infection is not well understood, and efforts to determine its significance have been limited. Future studies in disease-endemic areas are needed to determine if asymptomatic infections may play a role in population immunity (herd immunity) against leptospirosis.

We found that the presence of more than two wounds remained independently associated with infection, while wearing trousers or long skirts was associated with protection against *Leptospira* infection in our multivariable model. This finding suggests that more than two wounds and the use of trousers or long skirts were the strongest independent predictors and protective factors for infection. The protective effect of the use of trousers or long skirts may be essential for intervention planning in Thailand. While previous studies have suggested the importance of broken skin in infection with leptospirosis, this study is the first that we are aware of to quantify the effect of skin wounds and suggest that risk may increase with increasing number of breaks in the skin (11,12). The location of a lesion was not significantly associated with infection in multivariable analysis, and data regarding severity of the skin wound were not collected. Broken skin probably facilitates the entry of *Leptospira* directly into the bloodstream and increases the number of bacteria that enter the host in a given exposure period.

These findings suggest that further education efforts are needed to encourage the practice of wearing protective clothing while working in areas of Thailand with endemic leptospirosis, and may have an application in the control of the nationwide epidemic. The significant association of more than two wounds with infection suggests that efforts are needed to reduce exposure to contaminated water when persons have open wounds are present. Our findings suggest that protective clothing and avoiding exposure to standing flood water by persons with open skin wounds may decrease the risk of leptospirosis in these settings.
